# Fungal keratitis caused by Beauveria bassiana: drug and temperature sensitivity profiles: a case report

**DOI:** 10.1186/1756-0500-7-677

**Published:** 2014-09-27

**Authors:** Arisa Mitani, Atsushi Shiraishi, Hitoshi Miyamoto, Atsuko Sunada, Akiko Ueda, Seishi Asari, Xiaodong Zheng, Yasuaki Yamamoto, Yuko Hara, Yuichi Ohashi

**Affiliations:** Department of Ophthalmology, Ehime University Graduate School of Medicine, Shitsukawa, Toon, Ehime, 791-0295 Japan; Department of Stem cell Biology, Shitsukawa, Toon, Ehime, 791-0295 Japan; Department of Infectious Diseases Medicine, Ehime University Graduate School of Medicine, Shitsukawa, Toon, Ehime, 791-0295 Japan; Department of Clinical Laboratory, Ehime University Hospital, Shitsukawa, Toon, Ehime, 791-0295 Japan; Department of Clinical Laboratory, Osaka University Hospital, Suita, Osaka Prefecture, 565-0871 Japan

**Keywords:** Fungal keratitis, Beauveria bassiana, In vivo confocal microscopy, Drug-sensitivity, Temperature sensitivity growth

## Abstract

**Background:**

*Beauveria bassiana* is an entomopathogenic fungus and is a rare cause of keratitis. We present a case of fungal keratitis caused by *B. bassiana* that was diagnosed by in vivo confocal microscopy and in vitro corneal cultures. In addition, we determined the temperature- and drug-sensitivities of the isolated strain of *B. bassiana*.

**Case presentation:**

A 59-year-old Japanese man with a 2-month history of keratitis was examined by slit-lamp biomicroscopy, in vivo confocal microscopy, and histology and cultures of corneal scrapings. The corneal scrapings were used to determine the minimal inhibitory concentrations of different antifungal drugs and also to determine the temperature-sensitivity. In vivo confocal microscopy and histological examinations showed filamentous fungal keratitis. The characteristics of the fungal growth indicated that the keratitis was caused by *B. bassiana*. The keratitis responded poorly to systemic and topical voriconazole and to natamycin ointment. However, it was resolved after changing the natamycin to micafungin combined with surgical debridement. The isolated strain was sensitive to itraconazole, miconazole, micafungin, voriconazole, and resistant to flucytosine and fluconazole. It was moderately sensitive to amphotericin B, and natamycin. After 7 days in culture, the isolate grew small white colonies at 25°C, very small colonies at 35°C and 37°C.

**Conclusion:**

The drug-sensitivity and temperature-sensitivity profiles of *B. bassiana* should be helpful in the treatment of *B. bassiana* keratitis. Therapeutic surgery may be helpful for mycotic keratitis poorly responsive to medical therapy alone.

## Background

*Beauveria bassiana* is a fungus that is distributed worldwide. It can be isolated from soil, insects, and mites. It is an entomopathogenic fungi and is used as a biological control agent for insect pests as alternative supplements to chemical insecticides [1–3]. Although *B. bassiana* is considered to be non-pathogenic to vertebrates, a small number of patients with keratitis caused by *B. bassiana* have been reported [4–13]. We present a case of fungal keratitis caused by *B. bassiana* that was diagnosed by in vivo confocal microscopy and in vitro corneal cultures. In addition, we determined the temperature- and drug-sensitivities of the isolated strain of *B. bassiana*.

## Case presentation

The patient was a 59-year-old Japanese man who noted a corneal opacity in his right eye in October, 2009. However, he did not seek treatment for the opacity for about two months. He visited a private ophthalmological clinic on December 11, 2009, because his right eye felt irritated and his vision had decreased. The patient was a farm worker, but did not use entomopathogenic fungi as a biological control agent. He had no systemic diseases and no signs or symptoms of immunosuppression during the clinical course. He was diagnosed with infectious keratitis and was treated with topical gatifloxacin and erythromycin eye ointment for two weeks. Because the signs and symptoms did not improve, he was referred to the Ehime University Hospital on December 28, 2009.

**Figure 1:**
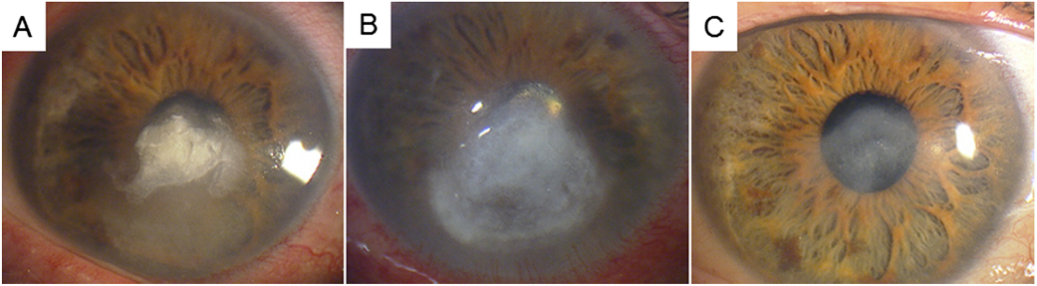
**Slit-lamp photographs of the clinical course of** ***Beauveria bassiana*** **keratitis** ***.*** **A**. Slit-lamp photograph taken at the initial examination showing a grayish stromal infiltrate with a dry texture and indistinct margins. **B**. Slit-lamp photograph 1 month after antifungal therapy with no improvement in the grayish stromal infiltrate. **C**. Slit-lamp photograph 10 month after treatment showing a scar with no infiltration into the stroma.

**Figure 2:**
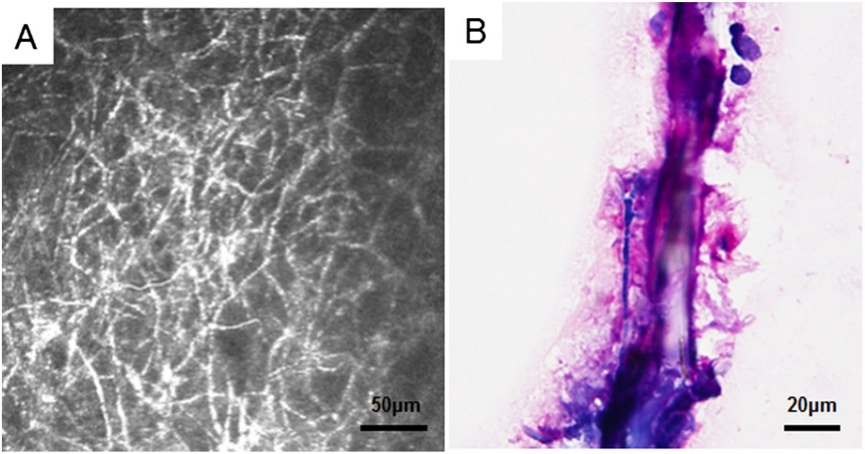
**In vivo confocal microscopic image of** ***Beauveria bassiana*** **keratitis. A**. A mass of interlocking and branching white lines in the area of the infiltrate indicating filamentous fungi. **B**. Microscopic examination of the corneal scrapings showing filamentous fungal hyphal fragments.

On the initial examination, his best-corrected visual acuity was 20/500 OD and 20/16 OS. Slit-lamp examination of the right eye showed a grayish stromal infiltrate with a dry texture and indistinct margins (Figure 1A). Examination of the cornea with in vivo confocal microscopy (HRT II-RCM; Heidelberg Engineering, Heidelberg, Germany) showed a mass of branching and interlocking white lines in the area of the infiltrate suggesting filamentous fungus keratitis (Figure 2A). Microscopic examination of corneal scrapings showed filamentous fungal hyphal fragments (Figure 2B).

Treatment was begun with oral voriconazole (400 mg/day) p.o., topical 0.1% voriconazole hourly, and natamycin eye ointment 5 times/day. Because the lesion did not respond despite the intensive antifungal therapy, the isolate from the corneal scrapings was sent to the First Laboratory in Medical Mycology Research Center, Chiba University for identification and to determine the minimal inhibitory concentrations (MICs) to different antifungal drugs (Table 1).

**Table 1:** **Minimal inhibitory concentrations of different antifungal drugs**

Drug	RPMI	0.5% Glucose-YNB
AMPH	0.5	4
5-FC	>64	>64
FLCZ	4	64
ITZ	0.03	0.03
MCZ	0.125	0.25
MCFG	0.125	0.06
VCZ	0.25	0.5
		MIC (μg/ml)

RPMI; Roswell Park Memorial Institute medium, YNB; yeast nitrogen base.

AMPH; amphotericin B, 5-FC; flucytosine, FLCZ; fluconazole, ITZ; itraconazole, MCZ; miconazole, MCFG; micafungin, VCZ; voriconazole, MIC; minimal inhibitory concentration.

**Figure 3:**
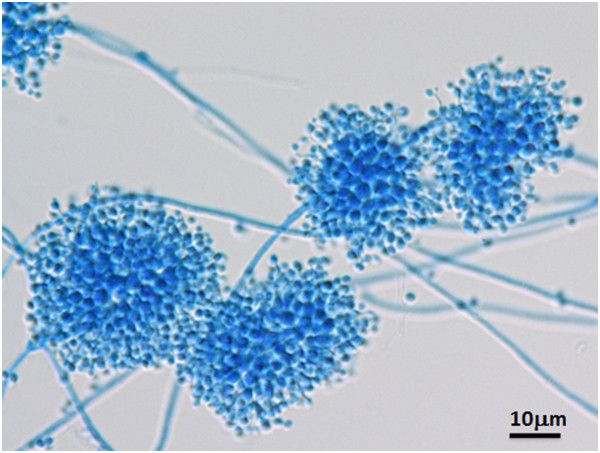
**Microscopic examination of the culture media.** Microscopic examination of the culture media stained with lactophenol-cotton blue shows conidia in distinctive blue spore balls composed of clusters of conidiogenous cells. The conidiogenous cells are short and ovoid and terminate in a narrow apical extension called a rachis. The rachis elongates after each conidium is produced resulting in a long zig-zag extension. These features are characteristic of *B. bassiana*.

The corneal scrapings grew fungal colonies on Sabouraud dextrose agar and white colonies on potato dextrose agar (Figure 3A). Microscopic examination of the culture media stained with lactophenol cotton blue showed conidia in distinctive blue spore balls composed of a cluster of conidiogenous cells. The conidiogenous cells were short and ovoid and terminated in a narrow apical extension or rachis. The rachis was elongated after each conidium resulting in a long zig-zag extension. These features are characteristic of *B. bassiana* (Figure 3B).

The isolate was confirmed to be *B. bassiana* by DNA sequencing at the First Laboratory in Medical Mycology Research Center, Chiba University. The MIC of natamycin was determined to be 1 μg/ml by the modified agar plate dilution method at the Department of Infection Control, Osaka University Hospital [14–16].

After receiving the MIC results, the natamycin eye ointment (5 times/day) was changed to topical 0.1% micafungin (hourly) along with topical 0.1% voriconazole. During the next 3 months, surgical debridement was also performed 3 times and the infectious lesion slowly improved. Finally, the infectious lesion healed but with mild scar formation (Figure 1C). His best-corrected visual acuity in his right eye improved to only 20/100 because of the corneal opacity and irregular astigmatism due to the surgical debridement.

**Figure 4:**
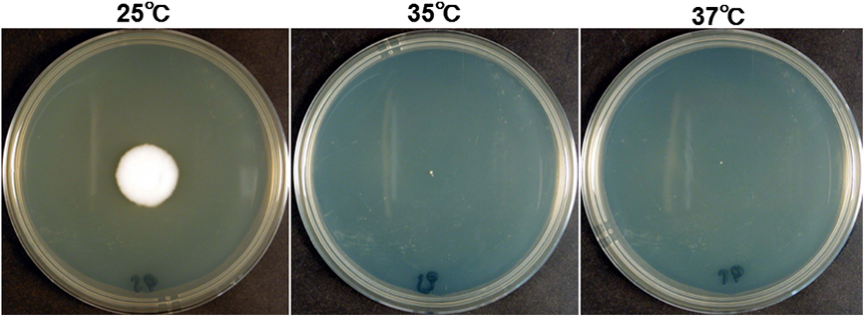
**Fungal colonies grown on Sabouraud dextrose agar at different temperatures.** The isolate grew small white colony at 25°C, very small colony at 35°C, and 37°C after 7 days. (n = 3)

To try to determine the cause of the slow clinical course and poor response to antifungal agents, the isolate was grown on Sabouraud dextrose agar at 25°C, 35°C, and 37°C for up to 7 days at the Clinical Laboratory of Ehime University Hospital as described in detail [14]. The isolate grew very slowly, and no determination could be made at 5 days when most filamentous fungi would have already grown colonies. At 7 days, the isolate grew small white colonies at 25°C, and very small colonies at 35°C and 37°C (Figure 4).

## Discussion

The entomopathogenic fungi belonging to the genus *Beauveria* are rare pathogens in humans, and only 11 cases of keratitis due to *Beauveria* species have been reported [4–13]. Because of the limited number of cases, the clinical features of *B. bassiana* keratitis have not been determined. The course of our non-necrotic keratitis case was slow-growing, there was no clinical improvement after treatment with voriconazole even though voriconazole was the preferable antibiotic according to the sensitivity tests. The cases of *B. bassiana* reported by Low *et al.*, Kisla *et al.*, and Tu *et al.*, were also slow-growing with late penetration into the corneal stroma which is consistent with our case [6, 7, 12]. In addition, the resistance to antifungal therapy was also consistent with our case [6–8, 12].

Our antifungal susceptibility tests showed that the isolated strain was sensitive to micafungin and voriconazole and moderately sensitive to natamycin. There have been several reports on the drug sensitivity of *B. bassiana*. Tu *et al.* and Figueira *et al.* reported that both of their strains were sensitive to posaconazole and voriconazole but resistant to amphotericin. These sensitivities are similar to that of our strain of *B. bassiana* [5, 12]. The results of Sonoyama *et al.* showed that the drug sensitivity of their *Beauveria* species was similar to our strain [11]. In addition, voriconazole was ineffective in both cases. Otherwise, the clinical course of the two cases was very similar, viz., slow-growing, non-necrotic keratitis, and clinically resistant to voriconazole. However, the causative strain was sensitive to voriconazole. Sonoyama *et al.* also had a successful treatment with topical voriconazole, although they also treated the eye with topical miconazole and oral itraconazole which their strain was sensitive to [11].

The results of the temperature sensitivity profile supported the microbiological results reported by Figueira *et al.* [5]. The temperature in the anterior chamber of the eye is generally 35°C or higher, and that of the corneal surface is 35°C or lower depending on the ambient temperature [17, 18]. Thus, we suggest that the *B.* bassiana strain in our case could not grow at temperatures of 35°C or higher and thus did not penetrate into the corneal stroma. This temperature sensitivity might be one reason why *B. bassiana* is a rare pathogen in humans, and why the infections in humans are limited to the body surface such as the cornea.

Another reason for the infection of the cornea may be the ability of *B. bassiana* to produce chitinase which lyses not only chitin but also keratin and collagen. This would then enable it to adhere and penetrate the cornea [5, 12]. *B. bassiana* has 3 different cell forms and can adhere to both hydrophobic and hydrophilic surfaces. This would enhance its adherence to the corneal surface along with the permissive avascular immune environment of the central cornea [5, 12]. In addition, the slow-growing property may be one of the reasons why the initial intensive antifungal therapy seemed to be clinically ineffective, because voriconazole works by preventing fungi from producing ergosterol which is a component of fungal cell membranes, and ergosterol is necessary for fungi to proliferate. Thus, the initial treatment with voriconazole only appeared to be ineffective in such slow-growing fungi. Although the clinical course was very slow, the infectious lesion improved after changing the treatment from natamycin eye ointment to topical micafungin and surgical debridement.

Micafungin is a echinocandins which blocks fungal cell wall beta-glucan synthesis, thus the different actions of voriconazole and micafungin may have a synergetic effect. It is also possible that the surgical debridement might have worked in our case because it removed antigenic and infectious elements and necrotic tissues. This supports the results of earlier studies that surgical debridement or keratectomy is required for mycotic keratitis that respond poorly to medical therapy [19, 20]. Further experiments on animals may be helpful in testing this hypothesis.

## Conclusions

We report a case of *B. bassiana* keratitis. The drug-sensitivity and temperature sensitivity profiles of *B. bassiana* should be helpful in diagnosing and treating *B. bassiana* keratitis. Therapeutic surgery may also be helpful for mycotic keratitis poorly responsive to medical therapy.

## Consent

Written informed consent was obtained from the patient for publication of this Case Report and any accompanying images. A copy of the written consent is available for review by the Editor-in-Chief of this journal.

## Abbreviations

B. bassiana: Beauveria bassiana
MIC: Minimal inhibitory concentration
RPMI: Roswell Park Memorial Institute medium
YNB: Yeast nitrogen base
AMPH: Amphotericin B
5-FC: Flucytosine
FLCZ: Fluconazole
ITZ: Itraconazole
MCZ: Miconazole
MCFG: Micafungin
VCZ: Voriconazol.
